# A comparative study of the sarcopenia screening in older patients with interstitial lung disease

**DOI:** 10.1186/s12890-022-01840-3

**Published:** 2022-01-25

**Authors:** Masatoshi Hanada, Noriho Sakamoto, Hiroshi Ishimoto, Takashi Kido, Takuto Miyamura, Masato Oikawa, Hiroki Nagura, Rina Takeuchi, Yurika Kawazoe, Shuntaro Sato, S. Ahmed Hassan, Yuji Ishimatsu, Hideaki Takahata, Hiroshi Mukae, Ryo Kozu

**Affiliations:** 1 Department of Rehabilitation Medicine, Nagasaki University Hospital, 1-7-1 Sakamoto, Nagasaki, 852-8501 Japan; 2 Department of Physical Therapy Science, Nagasaki University Graduate School of Biomedical Sciences, Nagasaki, Japan; 3 Department of Respiratory Medicine, Nagasaki University Graduate School of Biomedical Sciences, Nagasaki, Japan; 4 Clinical Research Center, Nagasaki University Hospital, Nagasaki, Japan; 5 Institute of Health, Policy, Management and Evaluation, University of Toronto, Toronto, Canada; 6 Department of Nursing, Nagasaki University Graduate School of Biomedical Sciences, Nagasaki, Japan

**Keywords:** Interstitial lung diseases, Physical performance, Sarcopenia, Frailty

## Abstract

**Background:**

The Asian Working Group for Sarcopenia 2019 (AWGS 2019) is the gold standard diagnostic criteria for sarcopenia in Asian populations. The calf circumference (CalF), the strength, assistance in walking, rising from a chair, climbing stairs, and falls (SARC-F) and the SARC-CalF questionnaires for sarcopenia screening have been used by AWGS 2019. The aim of this study was to assess accuracy of these three sarcopenia screening tools in patients with interstitial lung disease**.**

**Methods:**

In this cross-sectional study, stable patients with interstitial lung disease were enrolled. The SARC-F, SARC-CalF, and CalF, used in patients with interstitial lung disease, were compared to the diagnostic criteria proposed by AWGS 2019. The accuracy of screening tools was compared using sensitivity and specificity. Moreover, areas under the receiver operating characteristic curves (AUC) were computed.

**Results:**

Seventy eight patients were analyzed, and sarcopenia was identified in 25 (32.1%) patients with interstitial lung disease by the AWGS 2019 criteria. The sensitivity of the CalF was highest (96%) of the three screening tools, while the specificity was 60%. The sensitivity of SARC-F and SARC-CalF were 24% and 68%, while the specificity were 92% and 66%, respectively. The AUCs of CalF, SARC-F, and SARC-CalF in all patients were 0.78, 0.58, and 0.67, respectively.

**Conclusions:**

The CalF is most suitable for screening sarcopenia in patients with interstitial lung disease, while SARC-F and SARC-CalF are not.

## Background

Aging is associated with increased susceptibility to a variety of chronic diseases including lung pathologies, such as ILD [1]. Milne et al. have pointed out that frailty is highly prevalent in ILD patients [2]. Frailty and sarcopenia result in a loss of functional independence, where sarcopenia may be a risk factor for frailty [3, 4]. Sarcopenia has been defined as a progressive and generalized skeletal muscle disorder that involves the accelerated loss of muscle mass and function, and increased adverse outcomes including falls, functional decline, frailty, and mortality [5, 6]. The relationship between sarcopenia and adverse outcomes has been reported in various clinical populations [7–9]. In brief, sarcopenia is directly linked to activity limitation and is a huge concern for older patients. Despite knowledge of these risk factors, evaluation of sarcopenia is not common and little is known about its epidemiology in older patients with ILD [10].

Sarcopenia diagnosis and treatment were defined for Asians in the Asian Working Group for Sarcopenia (AWGS) 2014 consensus, and updated by the AWGS 2019 consensus [11]. In AWGS 2019, either calf circumference (CalF), the strength, assistance in walking, rising from a chair, climbing stairs, and falls (SARC-F) questionnaire or SARC-F combined with CalF (SARC-CalF) questionnaire were utilized for case finding.

The SARC-F was proposed in 2013, and is regarded as a simple questionnaire [12]. This questionnaire is also used in case finding in European Working Group on Sarcopenia in Older People (EWGSOP) 2 [5]. This questionnaire offers high specificity to diagnose sarcopenia, but poses the issue of low sensitivity. Moreover, several studies have pointed out that the SARC-F underestimates prevalence [13, 14], which, however, can be measured more accurately with the SARC-CalF that adds a measure of CalF to SARC-F [15]. Important to note, ILD patients experience dyspnea with exertion and SARC-F that includes factors such as activity limitation through exertion may not be suitable for screening of chronic respiratory diseases including ILD. The most suitable choice to evaluate sarcopenia for chronic respiratory diseases with dyspnea between SARC-F, the SARC-CalF and CalF is unknown.

The present study aimed to assess physical performance screening tools for sarcopenia in patients with ILD including the SARC-F, SARC-CalF and CalF in comparison to the diagnostic criteria proposed by the AWGS 2019. Additionally, we aimed to determine the most effective tool to assess sarcopenia.

## Methods

### Study design

This was a prospective, cross-sectional observational study that enrolled ILD patients ≥ 60 years of age from January 2020 to May 2021. Subjects gave their written, informed consent, and the study was approved by the Human Ethics Review Committee of Nagasaki University Hospital (approval number: 19121610).

### Subjects

Patients with ILD, including idiopathic interstitial pneumonias (IIPs), connective tissue disease-associated interstitial pneumonia, and hypersensitivity pneumonitis were recruited at the Department of Respiratory Medicine, Nagasaki University Hospital. Diagnostic criteria for IIPs and hypersensitivity pneumonitis were consistent with the International Consensus Statement [16]. Subjects were included if they were under the care of a respiratory physician, were ambulant, and were clinically stable with no changes in medication for at least four weeks before enrollment. Exclusion criteria were comorbid conditions affecting exercise performance (e.g., musculoskeletal or neurological disorders,), severe cognitive impairment, pregnancy, recent thoracic surgery, and active cancer treatment.

## Measurement

### ILD-GAP model

The interstitial lung disease-gender, age and lung physiology (ILD-GAP) model was created by adding the ILD subtype variable to the original GAP model [17]. The two lung physiology variables in this model include forced vital capacity (FVC) and diffusion capacity for carbon monoxide (DLco). Points were assigned for each variable to obtain a total point score (range 0–8). Demographic and clinical information including physical function, biochemistry of blood and pulmonary function test results were obtained from medical records. Subjects were divided into two groups: low ILD-GAP group (ILD GAP score 0‒2) and high ILD-GAP group (ILD GAP score 3‒8) [18, 19]. The results of the three screening methods were compared in each group.

### The SARC-F Questionnaire, the SARC-CalF Questionnaire, and CalF

**Fig. 1:**
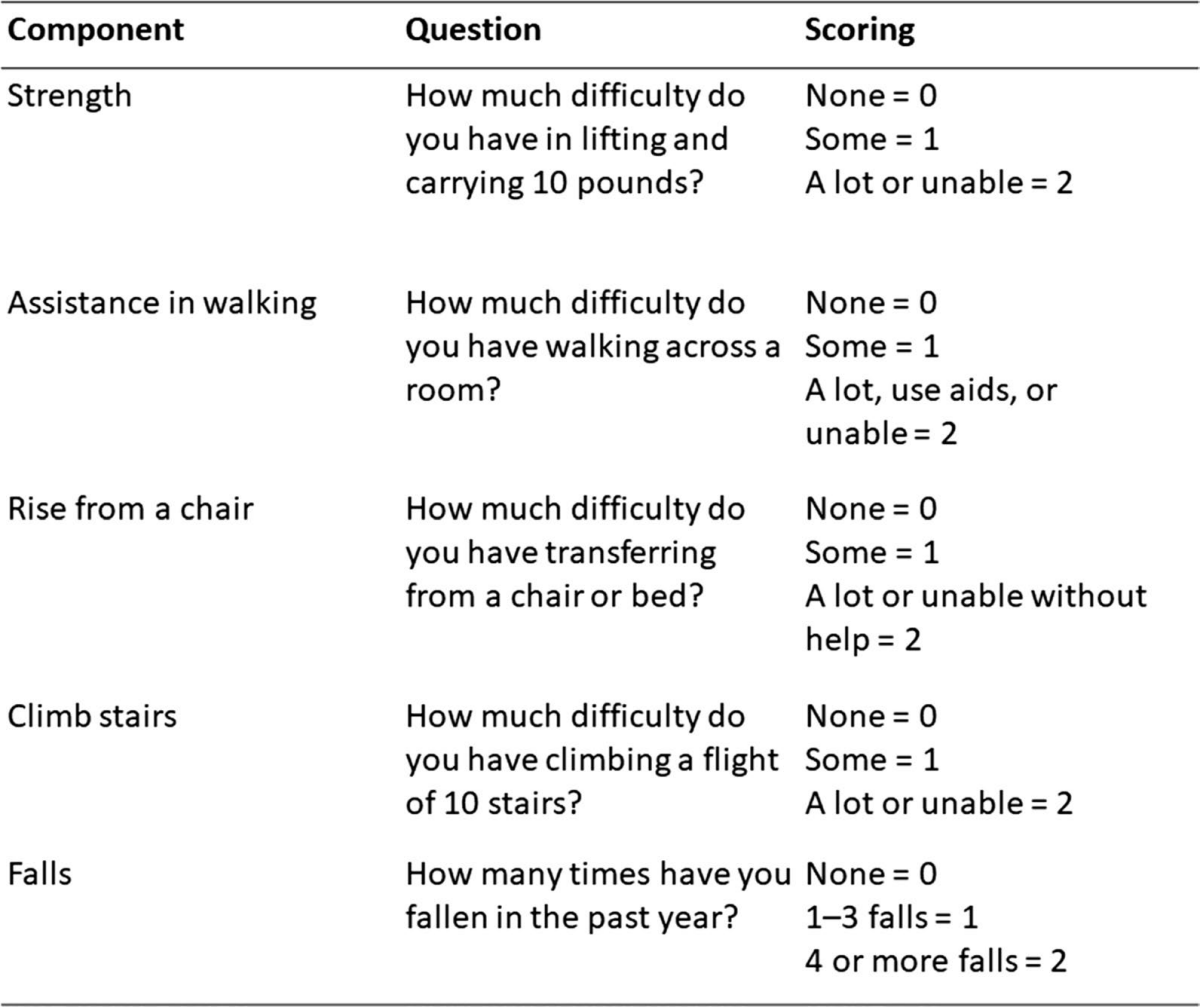
The SARC-F questionnaire for sarcopenia. The SARC-F: the strength, assistance in walking, rising from a chair, climbing stairs, and falls

The SARC-F Questionnaire was used to measure probable sarcopenia. The SARC-F questionnaire for sarcopenia is shown in Fig. 1 [12]. This questionnaire is composed of five items: strength, assistance in walking, rising from a chair, climbing stairs, and falls [12]. The SARC-F scores range from 0 to 10, with 0 to 2 points for each component [scoring range: 0 (best) to 10 (worst)]. Patients with a total score ≥ 4 were classified as having a risk of sarcopenia [20]. The SARC-CalF questionnaire is a combination of calf circumference and SARC-F, and a score of ≥ 11 points indicates probable sarcopenia. The maximum CalF was measured in a sitting position with hip and knee joint flexed at approximately 90°. CalF was measured at the point of the largest circumference. Calf < 34 cm for males and < 33 cm for females indicated sarcopenia. The CalF item is scored as 0 points if the CalF is > 34 cm for males and > 33 cm for females and 10 points if the calf circumference is ≤ 34 cm for males and ≤ 33 cm for females [15].

### Diagnostic criteria for sarcopenia

**Fig. 2:**
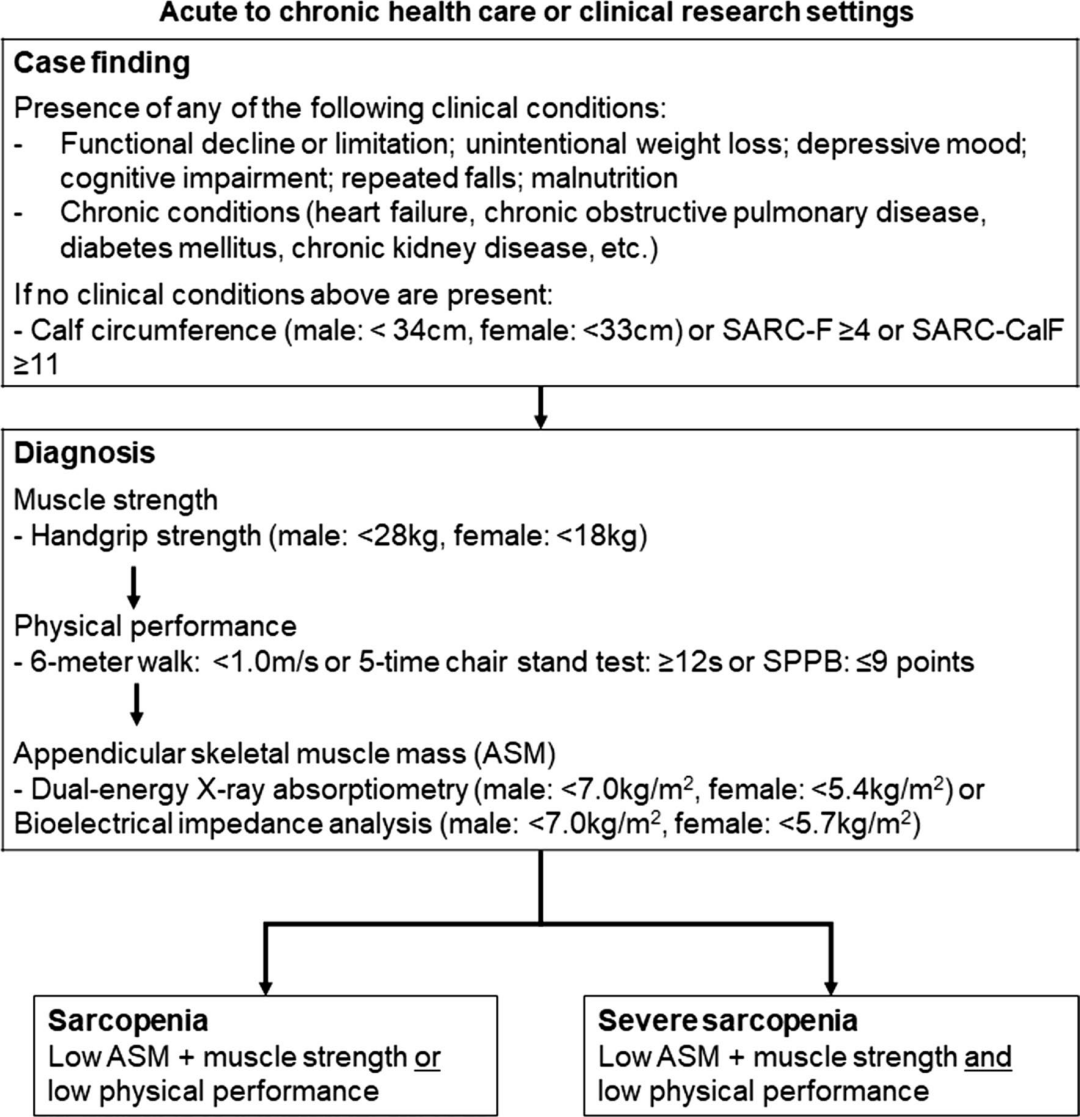
The diagnostic criteria for sarcopenia used by the Asian Working Group for Sarcopenia 2019. The SARC-F: the strength, assistance in walking, rising from a chair, climbing stairs, and falls; the SARC-CalF: the SARC-F combined with calf circumference; SPPB: short physical performance battery

Since the participants were older Japanese patients, they were evaluated according to the AWGS 2019 criteria of sarcopenia [11]. The diagnostic criteria for sarcopenia as used in AWGS 2019 is shown in Fig. 2. Estimation of sarcopenia at the first stage requires measurement by three screening methods: CalF, SARC-F and SARC-CalF. The second stage involves measurement of gait speed, grip strength, and muscle mass. Based on the AWGS 2019, low muscle strength is defined as handgrip strength < 28 kgf for males and < 18 kgf for females, while low physical performance is characterized as gait speed < 1.0 m/s. In addition, body composition and skeletal muscle mass were evaluated in ILD patients using the bioelectrical impedance analysis (BIA) method (InBody 270, InBody Japan, Tokyo, Japan). Low muscle mass diagnosis was defined as skeletal muscle index (SMI) < 7.0 kg/m^2^ for males and < 5.7 kg/m^2^ for females.

### Assessment of peripheral muscle force

Peripheral muscle force was evaluated via measurements of quadriceps and handgrip forces. Peak force developed during a maximal isometric knee extension was used as a measure of quadriceps force (QF). A hand-held dynamometer with a fixing belt (μ-Tas F-1; Anima Corporation, Tokyo, Japan) was used following a standard protocol [21]. The QF for the dominant side was tested in the sitting position with the hip and knee joints flexed at approximately 90°. The handgrip force (HF) was assessed in the dominant hand using a dynamometer (T.K.K.5401; Takei-Kiki-Kogyou Corporation, Niigata, Japan). HF was tested in sitting position with the elbow flexed at 90° and the arms fixed to the body. The average value of three attempts was recorded.

### The short physical performance battery (SPPB)

Physical performance was measured using the SPPB [22], which consists of three measures: walking speed, chair stands, and standing balance. A score from 0 (unable to complete the task) to 4 (best performance possible) was assigned to each measurement. The total score (0–12) was used to estimate overall physical performance level. Similar to previous studies [23–25], patients were separated into low physical performance-related risk (SPPB ≤ 9) or high physical performance-related risk (SPPB > 9).

### Functional exercise capacity

The 6-min walk test was performed based on published guidelines [26, 27]. The greater distance of two attempts was recorded. Oxygen saturation (SpO_2_) was monitored continuously throughout both tests (Konica Minolta Pulsox Me Oximeter, Osaka, Japan). If SpO_2_ decreased below 80%, the tests were terminated. Pre-exercise SpO_2_ and the lowest SpO_2_ during the tests were recorded.

### Activities of daily living

Activities of daily living (ADL) were evaluated using the Kats basic ADL scale [28]. The scale evaluates activities including feeding, ability to transfer, dressing, bathing, shopping and transportation. For each of the 6 activities, a score of 0 (dependent) or 1 (independent) was assigned. The sum of scores was used to obtain a measure of ADL performance.

### Health-related quality of life

Health-related quality of life (HRQL) was evaluated using the King's Brief Interstitial Lung Disease (K-BILD) health status Japanese version questionnaire [29]. The K-BILD is an ILD-specific HRQL questionnaire that measures health impairment. It is comprised of 15 items in three domains: breathlessness and activities, chest symptoms, and a psychological health. The K-BILD score ranged from 0 to 100, where higher values indicated better health.

### Statistical analysis

Baseline characteristics were summarized with frequencies and percentages for categorical data, while means and standard deviations for continuous data. The Shapiro–Wilk test was used to examine data distribution. Comparisons between sarcopenia and non-sarcopenia groups were made with unpaired *t*-tests, Mann–Whitney U tests, or Fisher's exact test. To evaluate the accuracy of screening by SARC-F, the SARC-CalF, and CalF, sensitivity, specificity, positive predictive value (PV+) and negative predictive value (PV−) were calculated for each screening method. The gold standard for calculating sensitivity and specificity was set at AWGS 2019 criteria. ILD-GAP model was used to evaluate the applicability of the three screening methods to ILD patients. To compare the accuracy among the screening tools, receiver operating characteristic curves (ROC) were constructed and area under ROC curves (AUC) were calculated. In addition, Spearman's rank correlation coefficient was used to examine relationship of K-BILD with patient-reported outcomes (SARC-F, SARC-CalF and CalF). The level of significance was 0.05 for all statistical tests. All statistical analyses were performed using JMP 15.0 software (SAS Institute Japan, Tokyo, Japan).

## Results

### Patients’ characteristics

The baseline characteristics of the 78 subjects are presented in Table 1. The mean age of patients was approximately 71 years. In the sarcopenia and non-sarcopenia group, patients had diagnoses of idiopathic pulmonary fibrosis (n = 10, n = 31), IIPs other than IPF (sarcopenia group n = 12: crypto-genic organizing pneumonia n = 3, pleuroparenchymal fibroelastosis n = 2, Unclassifiable n = 7, non-sarcopenia group n = 12: nonspecific interstitial pneumonia n = 1, lymphoid interstitial pneumonia n = 1, combined pulmonary fibrosis and emphysema n = 1, interstitial pneumonia with autoimmune features n = 2, Unclassifiable n = 6), connective tissue disease-associated interstitial pneumonia (n = 9: Sjogren syndrome n = 3, Mixed Connective Tissue Disease n = 1, Rheumatoid arthritis n = 3, pleuroparenchymal fibroelastosis n = 2), and hypersensitivity pneumonitis (n = 2, n = 2), respectively. The number of patients treated corticosteroid was not significantly different between sarcopenia group and non-sarcopenia group (Table 1). Furthermore, the duration (median (IQR)) of diagnosis to enrollment in patients receiving corticosteroid in sarcopenia group was 2 (1–14) months and 3 (1–18) months in the non-sarcopenia group. These results were not significantly different between groups. Type of ILD patients receiving corticosteroid were CHP (n = 2), CVD-IP (n = 4), COP (n = 2) and unclassified (n = 4). Pulmonary function tests were similar in both groups. The HF and QF as peripheral muscle force were not significantly different between groups with and without sarcopenia. The CalF, SMI, SARC-F and the SARC-CalF were not significantly different between sarcopenia and non-sarcopenia groups, separately. All patients were independent according to the ADL score. In HRQL, the total score of K-BILD questionnaire was within the average range 50–60% for both groups.

**Table 1:** Patients’ characteristics

	Overall (n = 78)	AWGS 2019 criteria classification		
		Sarcopenia (n = 25)	Non-sarcopenia (n = 53)	*P *value
Age, years	71 (67–77)	72 (68–77)	71 (67–82)	0.499
Sex male, %	51 (65)	16 (64)	35 (66)	1.00
BMI, kg/m^2^	24 (20–26)	23 (20–28)	24 (22–26)	0.855
Diagnosis, IIPs/other ILDs	49/29	15/10	34/19	0.804
Duration from diagnosis, months	3 (1–16)	2 (1–12)	6 (1–23)	0.227
Corticosteroid, %	12 (15)	6 (24)	6 (11)	0.184
Antifibrotic drugs, %	16 (21)	4 (16)	12 (23)	0.564
Long-term oxygen therapy, %	11 (14)	5 (20)	6 (11)	0.316
ILD-GAP score, point	3 (2–4)	3 (2–4)	3 (2–4)	0.551
mMRC dyspnea scale, grade	2 (1–2)	2 (1–2)	1 (1–2)	0.754
PaCO_2_ at rest, mmHg	41 (38–46)	40 (38–44)	42 (38–51)	0.417
PaO_2_ at rest, mmHg	80 (63–91)	86 (73–94)	75 (61–91)	0.219
FVC, %	85 (68–97)	85 (63–93)	85 (72–98)	0.316
FEV_1_, %pred	90 (75–101)	90 (69–103)	90 (77–101)	0.863
FEV_1_/FVC, %	78 (72–86)	80 (72–87)	78 (72–85)	0.514
DL_CO_, %	64 (45–80)	70 (45–83)	60 (45–80)	0.271
KL-6, U/ml	715 (451–1165)	662 (474–1168)	740 (407–1155)	0.768
HF, kgf	26 (20–33)	23 (19–33)	27 (22–34)	0.167
QF, kgf	28 (19–35)	23 (16–33)	29 (20–36)	0.169
Calf circumference, cm	33 (30–35)	33 (30–37)	34 (30–35)	0.996
SMI, kg/m^2^	7 (6–7)	7 (5–7)	7 (6–8)	0.201
SPPB, point	11 (11–12)	12 (11–12)	12 (11–12)	0.912
SARC-F, point	1 (0–2)	1 (0–4)	1 (0–2)	0.486
SARC-CalF, point	2 (0–11)	11 (0–12)	1 (0–11)	0.171
6MWD, m	424 (355–500)	392 (308–509)	442 (371–500)	0.317
ADL score	6 (6–6)	6 (6–6)	6 (6–6)	0.204
K-BILD, point	54 (47–64)	53 (44–68)	54 (48–62)	0.684
Psychological symptoms	51 (43–63)	49 (43–67)	52 (42–60)	0.720
Breathlessness and activities	44 (34–53)	40 (31–55)	46 (37–53)	0.232
Chest symptoms	73 (64–85)	73 (47–85)	73 (64–85)	0.779

Categorical variables were analyzed using Fisher's exact test, while continuous variables with unpaired t-tests and Mann–Whitney U test. Values are median (interquartile range) or numbers (percentage) of subjects

ADL score, activities of daily living score; BMI, Body Mass Index; CalF, calf circumference; DLCO, diffusion capacity for carbon monoxide; %FEV1, percentage of forced expiratory volume in one second; FVC, forced vital capacity; HF, handgrip force; IIPs, idiopathic interstitial pneumonias; ILD-GAP, interstitial lung disease-gender, age, lung physiology score; K-BILD, King’s Brief Interstitial Lung Disease, KL-6, Krebs von den Lungen-6; mMRC, Modified Medical Research Council; 6MWD, six-minute walking distance; PaCO2, partial pressure of carbon dioxide; PaO2, partial pressure of oxygen; %pred, percent predicted; QF, quadriceps force; the SARC-F, the strength, assistance in walking, rising from a chair, climbing stairs, and falls; SARC-CalF, SARC-F combined with calf circumference; SMI, skeletal muscle index; SPPB, short physical performance battery; %VC, percentage of volume capacity

### Prevalence of sarcopenia

In our patients, the prevalence of sarcopenia was 32.1% according to the AWGS 2019 criteria: males: 31.4%; females: 33.3%. There were no significant differences in the prevalence of sarcopenia between the two sexes. Overall, 24 (96%) patients screened positive for sarcopenia using CalF, 6 (24%) using SARC-F questionnaire, and 17 (68%) using SARC-CalF questionnaire based on the AWGS 2019 criteria. Significant negative correlations were found between K-BILD and SARC-F (r =  − 0.243, *p* = 0.033), but not SARC-CalF (r =  − 0.128, *p* = 0.267) or CalF (r = 0.137, *p* = 0.233).

### Sensitivity, specificity and predictive values of sarcopenia screening

**Fig. 3:**
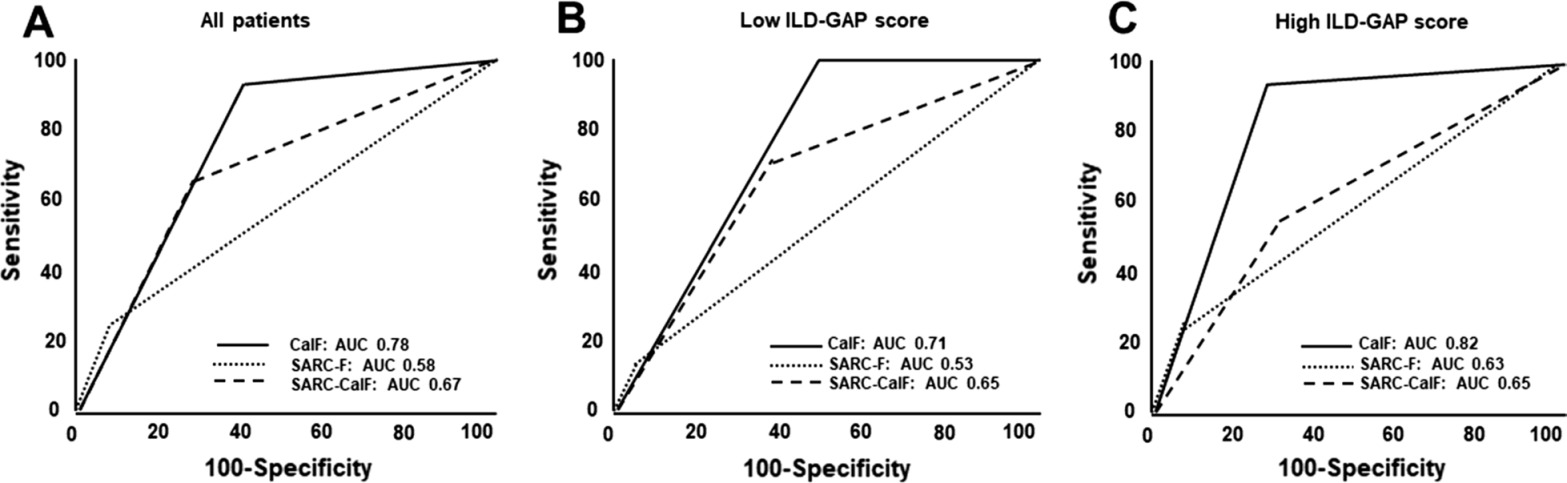
The ROC curves of three screening methods according to AWGS 2019. **A** All patients, **B** low ILD-GAP score group, **C** high ILD-GAP score group. AUC, area under the curve; CalF, calf circumference; ROC, receiver operating characteristic; the SARC-F, the strength, assistance in walking, rising from a chair, climbing stairs, and falls; the SARC-CalF, the SARC-F combined with calf circumference

The sensitivity of the SARC-F for the detection of case finding in sarcopenia was 24%; conversely, the specificity was 92% (Table 2). Overall, the sensitivity of the CalF was 96%, the highest amongst the three sarcopenia screening tools. The PV+ of all items were low whereas the PV- were relatively high (> 50%). The results of the sensitivity–specificity analysis were not affected by high or low ILD-GAP score groups (ILD severity). Furthermore, CalF provided better sensitivity than other items of SARC-F and SARC-CalF in patients with ILD (Table 3). The CalF alone had the best sensitivity (AUC) in screening sarcopenia compared to SARC-F and SARC-CalF according to AWGS 2019 criteria (Fig. 3).

**Table 2:** Sensitivity, specificity and predictive values of three sarcopenia screening in patients with ILD

	Sensitivity (%)	Specificity (%)	PV+	PV−
All patients				
Calf circumference	96.0	60.0	53.3	97.0
SARC-F	24.0	92.0	60.0	72.1
SARC-CalF	68.0	66.0	48.6	81.4
Low ILD-GAP score < 3				
Calf circumference	100	41.0	47.4	100
SARC-F	11.1	94.0	50.0	66.7
SARC-CalF	77.8	53.0	46.7	81.8
High ILD-GAP score ≥ 3				
Calf circumference	91.7	72.0	57.9	95.5
SARC-F	33.3	93.0	66.7	77.1
SARC-CalF	58.3	72.0	46.7	80.8

ILD, interstitial lung disease; ILD-GAP, interstitial lung disease-gender, age, lung physiology score; PV+, positive predictive value, PV−, negative predictive value; SARC-F, the strength, assistance in walking, rising from a chair, climbing stairs, and falls; SARC-CalF, SARC-F combined with calf circumference

**Table 3:** Sensitivity, specificity and predictive values of items of the SARC-F and calf circumference in screening for sarcopenia in patients with ILD

SARC-CalF	Positive screening n (%)	Sensitivity (%)	Specificity (%)	PV+	PV−
Strength	29 (34.5)	33.3	69.0	36.0	66.0
Assistance in walking	8 (9.5)	71.4	72.0	20.0	96.2
Rising from a chair	11 (13.1)	44.4	70.0	16.0	90.1
Climbing stairs	48 (57.1)	34.1	71.0	60.0	45.3
Falls	16 (19.0)	33.3	68.0	20.0	81.1
Calf circumference	48 (57.1)	100	51.0	50.0	100

ILD, interstitial lung disease; PV+, positive predictive value, PV−, negative predictive value; SARC-F, strength, assistance in walking, rising from a chair, climbing stairs, and falls; SARC-CalF, the SARC-F combined with calf circumference

## Discussion

The main findings of the present study are as follows: (1) in ILD patients with normal BMI and mild mMRC dyspnea scale, sarcopenia was identified in 25 (32.1%) patients by the AWGS 2019 criteria; (2) the CalF was the most sensitive in identifying sarcopenia compared to SARC-F and SARC-CalF. These screening tools showed a similar trend regardless of the ILD-GAP score (severity of ILD); (3) CalF items had better sensitivity in the SARC-CalF. To our knowledge, this is the first report comparing the accuracy of SARC-F, SARC-CalF, and CalF in screening sarcopenia in patients with ILD.

Sarcopenia was identified in 25 (32.1%) patients with ILD by the AWGS 2019 criteria. Although, previous studies have reported the sarcopenia prevalence of 5–25% in older population [11, 30–32], the present study indicated a higher prevalence. This highlights the importance of accurate sarcopenia screening to improve patient outcomes. Improvement in survival rate is expected in ILD patients if their disease progression is suppressed by treatment (e.g., antifibrotic drugs). However, it is speculated that aging associated sarcopenia may pose a problem [33]. Therefore, it is important to screen sarcopenia in patients with ILD as early as possible.

In the present study, the SARC-F had low sensitivity, high specificity, low PV+ and high PV−. These results are consistent with those in different populations [34–36]. The poor sensitivity reported in this study and several previous studies may suggest that SARC-F is not a suitable screening test for sarcopenia in ILD patients. The issue of underestimation of accuracy by SARC-F was resolved using SARC-CalF that adds a measure of CalF to SARC-F [13]. Mo et al. pointed out that low-to-moderate sensitivity of the SARC-F is not adequate for population wide screening [30]. Similar conclusion was drawn in this study with a small, yet specific group of ILD patients. Moreover, almost all questions in the SARC-F and SARC-CalF are affected by dyspnea through exertion except CalF. Therefore, it was speculated that the SARC-F and SARC-CalF are not suitable for screening sarcopenia in patients with chronic respiratory diseases including ILD. However, the SARC-F had the highest specificity in our results. Although the SARC-F may be an effective tool for selecting subjects who should undergo further testing for sarcopenia, careful attention should be paid to the fact that there could be multiple omissions when SARC-F is used for excluding sarcopenia [37]. In this study, we found significant negative correlation between K-BILD and SARC-F. Thus, although SARC-F may not be suitable for excluding sarcopenia, it has the potential to be a useful assessment tool to evaluate health-related quality of life. It should also be noted that in patients with respiratory diseases, the results from questionnaire are likely impacted by age and exertion on dyspnea, making it difficult to assess sarcopenia accurately. In any case, the reliability, validity and utility of SARC-F needs further investigation in patients with respiratory diseases. Conversely, the CalF measurement may be the best screening option as it is simple and convenient. The CalF takes only takes a few minutes to complete, requires little training to administer. In addition, the results can be quantified, are reproducible and sensitive to changes in functionality through time. Therefore, if we can diagnosis only by the CalF measurements to sarcopenia diagnosis, there are considerable benefits for ILD patients, it can be effectively used for early evaluation of sarcopenia in ILD patients. Better screening tools for sarcopenia would help identify frail patients and, thus, prompt more frequent referrals for pulmonary rehabilitation leading to health-related quality of life and exercise tolerance.

In SARC-CalF, CalF had better sensitivity than other items. Dyspnea during exertion is characteristic of chronic respiratory diseases including ILD. In this study, unlike previous studies that examined SARC-F items, the positive screening rate was highest for climbing stairs (57.1%) among the items that involve exertion [31]. The positive screening rate was high, but the sensitivity was low, making it difficult accurately screen for sarcopenia in ILD patients with only stair climbing component. Based on the results of this study, SARC-F and SARC-CalF are not suitable screening tool to assess sarcopenia in ILD patients. Therefore, development of a new sarcopenia screening tool for chronic respiratory diseases is desirable.

This study has several limitations. Firstly, the sample size was small and the study was a single-center trial. Larger, multicenter studies are needed to consider the development of a new sarcopenia screening tool. Secondly, in ILD patients excluding IPF, corticosteroids are one of the most common treatment options employed. However, corticosteroids decrease skeletal muscle function in ILD patients with mild symptoms [38] and are an independent risk factor for developing sarcopenia [39]. This suggests that the risk of developing sarcopenia is higher in ILD patients commonly treated by corticosteroids. Since our patients had numerous ILDs for which steroids are not prescribed, relationship between corticosteroid use and sarcopenia could not be evaluated using multivariable analysis. Larger studies are needed to consider the influence of corticosteroid use on developing sarcopenia in ILD patients. Thirdly, when using the SARC-F and the SARC-CalF to screen for sarcopenia according to the AWGS 2019 criteria, it is necessary to adjust the cut-off values that are suitable for chronic respiratory diseases. Finally, we used BIA for the assessment of SMI instead of CT, MRI or DEXA. Although, these methods are more precise, BIA prevents exposure to x-rays and provides a more efficient solution to measuring body composition and skeletal muscle mass. As a result, the AWGS 2019 criteria recommends BIA as an alternative option for muscle measurement.

## Conclusion

In conclusion, approximately 30% patients with ILD were found to have sarcopenia based on the AWGS 2019 criteria. Calf circumference had the best sensitivity out of the three screening tools and is, thus, most suitable for screening sarcopenia in patients with ILD. In the future, development of a new sarcopenia screening tool for chronic respiratory diseases should be considered.

## Data Availability

Not applicable.

## Abbreviations

ADL: Activities of daily living
AUC: Area under receiver operating characteristic curves
AWGS: The Asian Working Group for Sarcopenia
BIA: Bioelectrical impedance analysis
BMI: Body Mass Index
CalF: Calf circumference
DL_CO_: Diffusion capacity for carbon monoxide
EWGSOP: European Working Group on Sarcopenia in Older People
%FEV_1_: Percentage of forced expiratory volume in one second
FEV_1_/FVC: Forced expiratory volume in one second/forced vital capacity
HF: Handgrip force
HRQL: Health-related quality of life
IIPs: Idiopathic interstitial pneumonias
ILD: Interstitial lung disease
ILD-GAP model: Interstitial lung disease-gender, age, lung physiology
IPF: Idiopathic pulmonary fibrosis
K-BILD: King’s Brief Interstitial Lung Disease
mMRC dyspnea: Modified Medical Research Council dyspnea
6MWD: 6-Minute walking distance
PaCO_2_: Partial pressure of carbon dioxide
PaO_2_: Partial pressure of oxygen
PV+: Positive predictive value
PV−: Negative predictive value
%pred: Percent predicted
QF: Quadriceps force
ROC: Receiver operating characteristic curves
SARC-F: Strength, assistance in walking, rising from a chair, climbing stairs, and falls
SARC-CalF: SARC-F combined with calf circumference
SMI: Skeletal Muscle Index
SpO_2_: Oxygen saturation
SPPB: Short physical performance battery
%FVC: Percentage of forced vital capacity
